# Lung Cancer-Specific Mortality Risk and Public Health Insurance: A Prospective Cohort Study in Chongqing, Southwest China

**DOI:** 10.3389/fpubh.2022.842844

**Published:** 2022-04-29

**Authors:** Yuqi Wang, Haike Lei, Xiaosheng Li, Wei Zhou, Guixue Wang, Anlong Sun, Ying Wang, Yongzhong Wu, Bin Peng

**Affiliations:** ^1^Department of Epidemiology and Health Statistics, School of Public Health and Management, Chongqing Medical University, Chongqing, China; ^2^Chongqing Key Laboratory of Translational Research for Cancer Metastasis and Individualized Treatment, Chongqing University Cancer Hospital, Chongqing, China; ^3^MOE Key Lab for Biorheological Science and Technology, State and Local Joint Engineering Laboratory for Vascular Implants, College of Bioengineering Chongqing University, Chongqing, China

**Keywords:** lung cancer, public health insurance, prognosis, prospective cohort study, mortality risk

## Abstract

**Objective:**

The incidence and mortality of lung cancer rank first among malignant tumors, and its long treatment cycle will bring serious economic burdens to lung cancer patients and their families. There are few studies on the prognosis of lung cancer and insurance policies. This article explores the relationship between the lung cancer-specific death and public health insurance, self-paying rate, and the joint effect of public health insurance and self-paying rate.

**Materials and Methods:**

A prospective longitudinal cohort study was conducted in Chongqing, China from 2013 to 2019. The selected subjects were patients with C33–C34 coded according to the tenth edition of the International Classification of Diseases (ICD-10), aged 20 years or older. We conduct a subgroup analysis based on public health insurance types and self-paying rates. After following the inclusion and exclusion criteria, the *chi-square* test was used to describe the demographic and clinical characteristics of patients with different insurance types and different self-paying rates. Multivariate logistic regression was used to analyze the relationship between patients with different insurance types, self-paying rates, and lung cancer treatment methods. Finally, the Cox proportional hazard model and the competitive risk model are used to calculate the cumulative hazard ratio of all-cause death and lung cancer-specific death for different insurance types and different self-paying rate groups.

**Results:**

A total of 12,464 patients with lung cancer were included in this study. During the follow-up period (median 13 months, interquartile range 5.6–25.2 months), 5,803 deaths were observed, of which 3,781 died of lung cancer. Compared with patients who received urban resident-based basic medical insurance (URBMI), patients who received urban employee-based basic medical insurance (UEBMI) had a 38.1% higher risk of lung cancer-specific death (Hazard Ratios (HRs) = 1.381, 95% confidence interval (CI): 1.293–1.476, *P* < 0.005), Compared with patients with insufficient self-paying rate, patients with a higher self-paying rate had a 40.2% lower risk of lung cancer-specific death (HRs = 0.598, 95% CI: 0.557–0.643, *P* < 0.005). Every 10% increase in self-paying rate of URBMI reduces the risk of lung cancer-specific death by 17.6%, while every 10% increase in self-paying rate of UEBMI reduces the risk of lung cancer-specific death by 18.0%.

**Conclusions:**

The National Medical Security Administration should, under the condition of limited medical insurance funds, try to include the original self-paid anti-tumor drugs into the national medical insurance coverage. This can not only reduce the mortality rate of lung cancer patients, but also reduce the family burden of lung cancer patients. On the other hand, high-risk groups should increase their awareness of lung cancer screening and actively participate in the national cancer screening project led by the state.

## Introduction

As early as 2008, lung cancer replaced liver cancer as the most common cancer (1, 2), and it was also the leading cause of cancer death in China (3). Its incidence and mortality rate ranked first among malignant tumors (4). In 2011, according to a study conducted by the National Cancer Center (5), there were about 650,000 new cases of lung cancer each year, including about 440,000 men and 210,000 women. The crude incidence and mortality of lung cancer in China were 48.32/100,000 and 39.27/1,000,000 respectively. According to China's cancer registration data, the number of new lung cancer cases in China rose to approximately 787,000 and the number of deaths rose to 631,000 in 2015 (6). The incidence and mortality of lung cancer in Chongqing are higher than the national average. From 2010 to 2017, the incidence and mortality have increased by 63 and 26.46% respectively (7).

As a chronic lung disease, lung cancer has a long treatment cycle and complicated treatment conditions (8). Lung cancer treatment models and anti-cancer drugs are expensive, which not only brings heavy pressure to individuals and families, but also leads to a huge financial burden on the health system of a country, especially for developing countries where the health system that is not ready to alleviate the burden on everyone (1, 9, 10). The medical insurance system is a form of insurance used to compensate for the medical expenses consumed by the disease. By using the risk transfer and compensation transfer system in insurance science, the economic loss caused by the disease of a single patient is shared with other groups threatened by the same disease, and use the pooled medical insurance fund to make up and offset the economic losses caused by the disease (11). At present, most studies only focus on the relationship between insurance type and cancer prognosis, while few studies focus on the relationship between self-paying rate and cancer prognosis. Therefore, this study used a prospective large-scale cohort of lung cancer patients diagnosed in Chongqing from 2013 to 2019 years to explore the relationship between lung cancer specific mortality in Chongqing and their insurance types, self-paying rates, and the joint effects of insurance types and self-paying rates.

## Materials and Methods

### Data Source

This is a prospective cohort study based on the follow-up database of Chongqing University Cancer Hospital, which includes almost all lung cancer patients diagnosed in Chongqing since 2013.We selected 16,067 lung cancer patients from January 1, 2013 to December 31, 2019, and prospectively collected relevant demographics (gender, age at diagnosed, ethnicity, marital status, occupation, date of diagnosis) and clinical characteristics (cancer stage, pathological type), treatment methods (surgery, targeted therapy, immunity therapy, radiotherapy, chemotherapy) as well as follow-up information.

### Inclusion and Exclusion Criteria

Inclusion criteria for this study were (1) New-onset lung cancer patients diagnosed in this hospital; (2) The main treatment occurred in our hospital; (3) The patient's basic information, expenditure information, and clinical information (clinical diagnosis, treatment plan, pathological information) are complete. Exclusion criteria were (1) Non-new lung cancer patients, or no continuous treatment in this hospital. (2) missing important information such as insurance information and self-paying expense information; and (3) no follow-up records.

### Study Population Identification

The diagnosis of lung cancer patients included in the study was coded as C33–C34 according to the tenth edition of the International Classification of Diseases (ICD-10).

### Follow-Up Information

The patient's survival outcome information is obtained through a combination of active follow-up and passive follow-up, and the fundamental cause of death of the patient was from the medical records as much as possible, or informed by members of the immediate family. The date of the patient's first lung cancer diagnosis was used as the starting point for observed survival, and the time of death or the last follow-up was set as the end point. The follow-up period was in months and the follow-up time ended on December 31, 2019.

### Health Insurance

In 2003, China's urban basic medical insurance system mainly consisted of two public medical insurance systems, namely, urban resident-based basic medical insurance (URBMI) and urban employee-based basic medical insurance (UEBMI), making it easier for individuals to obtain medical services, aiming to achieve health coverage for the rural population across the country by 2010 (12–14). The current basic medical insurance system is set up according to the population. URBMI mainly covers the unemployed, children, and students; UEBMI mainly covers the urban employed population and retirees. There are great differences between URBMI and UEBMI in benefit level, reimbursement of serious illness insurance and treatment standards of outpatient chronic diseases (15). For example, the maximum payment limit of UEBMI is 260000 yuan, while the maximum payment limit of URBMI in the same insured year is 1,60,000 yuan. The total cost of this study refers to the total cost from the newly diagnosed lung cancer to the completion of all treatment, that is, the sum of all costs incurred during the observation period. The self-paying rate is defined as the medical expenses borne by the patient and their families divided by the total cost. Each person has a different self-paying rate for the same disease, which is affected by age, employment age, and disease treatment mode, and so on. Information about insurance types and self-paying rates in this study are recorded in the hospital information system (HIS). We classified patients by reimbursement rate below (0–49%) or above (50%-100%) the median.

### Lung Cancer-Specific and Overall Mortality

Active follow-up uses telephone, WeChat, Short Message Service (SMS) and other methods for follow-up, while passive follow-up uses outpatient, hospitalization and HIS to query and match the survival status of patients. We obtained the cause of death of patients by reporting the patient's death or actively following up with their family members. In this study, lung cancer-specific mortality was the primary outcome, and all-cause death was the secondary outcome.

In this study, lung cancer-specific mortality was the primary outcome, and all-cause death was the secondary outcome. The underlying cause of death of this study can be ascertained by medical records or can be informed by their immediate relatives through active follow-up.

### Statistical Analysis

Firstly, a descriptive analysis of baseline characteristics was performed. We used chi-square test to describe the demographic and clinical characteristics of patients with different insurance types and different self-paying rates, and count data were expressed as frequency and percentage. Adjusted according to different variables, multivariate logistic regression was used to respectively analyze the relationship between different insurance types, self-paying rate and treatment of the lung cancer (survey, targeted therapy, immunity therapy, radiotherapy, chemotherapy).

Secondly, in order to compare the risk of lung cancer-specific death and all-cause death of patients in the two insurance types and the two groups of self-paying rates. In the insurance type, we take the patients who receive URBMI as the reference, and the patients with low self-paying rate as the reference in the self-paying rate group, we use the Cox regression model to calculate the risk ratio and 95% confidence interval of lung cancer-specific death and all-cause death. In addition, we used the competitive risk model to calculate and plot the lung cancer specific death and all-cause death cumulative hazard.

Finally, in order to further understand the joint effect of insurance type and self-paying rate, we studied the association between every 10% increase in the self-paying rate and the risk of lung cancer-specific mortality, all-cause mortality.

In Model A, we adjusted for demographic factors, including age at diagnosis, sex, ethnicity, marital status, and occupation. In Model B, we also adjusted according to clinical characteristics, including the date of diagnosis of lung cancer, cancer stage, and pathological diagnosis. In Model C, we additionally adjusted for treatment types, whenever applicable, including surgery, targeted therapy, immunity therapy, radiotherapy, and chemotherapy. All variables are classified into the model. All data were analyzed using SAS9.4 statistical software (version 9.4; SAS Institute Inc., Cary, North Carolina) and software R (version 4.0.2; R Foundation for Statistical Computing, Vienna, Austria). The *P* < 0.05 value is statistically significant.

## Results

### Characteristics of Subjects

According to the inclusion and exclusion criteria, we excluded 3,534 patients with missing clinical information, 13 missing identity information, and 56 patients without insurance information and self-paying rates. In the final cohort, there were 12,464 lung cancer patients of which 8,757 were males (70.26%), and 3,707 were females (29.74%). They were basically Han and only 112 were minorities. Among the included study subjects, 6,496 received URBMI, and the remaining 5,968 received UEBMI. The median self-paying rate was 69% in URBMI, and 35% in UEBMI. After grouping self-paying rate, 5,156 patients had a self-paying rate of <50%, and the remaining 7,308 had a higher self-paying rate. The patients receiving URBMI were younger (60.11 ± 9.82 vs. 63.19 ± 10.54, *P* < 0.0001). In the different insurance type, there were no differences in marital status and date year at diagnosis, and the differences in other variables were statistically significant (*P* < 0.05). In the group of self-paying rate, except for the pathological diagnosis, and the other variables were statistically significant (*P* < 0.05). The detailed results are shown in Table 1.

**Table 1:** Characteristics of patients with lung cancer by insurance type and self-paying rate.

		**By insurance type**			**By self-paying rate**		
	**ALL(*N* = 12,464)** **N(%)**	**URBMI (*N* = 6,496) N(%)**	**UEBMI (*N* = 5,968)** **N(%)**	***P***	**0%-49%** **(*N* = 5,156)** **N(%)**	**50%-100%** **(*N* = 7,308)** **N(%)**	***P***
Age at diagnosis, years				<0.0001			<0.0001
18–39	231 (1.85)	109 (1.68)	122 (2.04)		71 (1.38)	160 (2.19)	
40–49	1,299 (10.43)	843 (12.98)	456 (7.64)		464 (9.00)	835 (11.43)	
≥50	10,934 (87.72)	5,544 (85.34)	5,390 (90.32)		4,621 (89.62)	6,313 (86.38)	
Sex				<0.0001			<0.0001
Male	8,757 (70.26)	4,400 (67.73)	4,357 (73.01)		3,957 (76.75)	4,800 (65.68)	
Female	3,707 (29.74)	2,096 (32.27)	1,611 (26.99)		1,199 (23.25)	2,508 (34.32)	
Ethnic group				<0.0001			0.0004
Han	12,352 (99.10)	6,417 (98.78)	5,935 (99.45)		5,128 (99.46)	7,224 (98.85)	
Minority	112 (0.90)	79 (1.22)	33 (0.55)		28 (0.54)	84 (1.15)	
Marital status				0.2455			0.0005
Married	11,670 (93.63)	6,098 (93.87)	5,572 (93.36)		4,874 (94.53)	6,796 (92.99)	
Unmarried/divorced/widowed and/or other	794 (6.37)	398 (6.13)	396 (6.64)		282 (5.47)	512 (7.01)	
Occupation				<0.0001			<0.0001
Company employees/workers and/or business units	1,581 (12.68)	752 (11.58)	829 (13.89)		768 (14.90)	813 (11.12)	
Self-employed/unemployed and/or freelancers	2,646 (21.23)	1,478 (22.75)	1,168 (19.57)		179 (3.47)	2,467 (33.76)	
Civil servants	106 (0.85)	13 (0.20)	93 (1.56)		67 (1.30)	39 (0.54)	
Other professional	8,131 (65.24)	4,253 (65.47)	3,878 (64.98)		4,142 (80.33)	3,989 (54.58)	
Date year at diagnosis				0.0942			<0.0001
2013–2016	3,403 (27.30)	1,732 (26.66)	1,671 (28.00)		2,100 (40.73)	1,303 (17.83)	
2017–2020	9,061 (72.70)	4,764 (73.34)	4,297 (72.00)		3,056 (59.27)	6,005 (82.17)	
Cancer stage				<0.0001			<0.0001
I	1,421 (11.5)	565 (8.70)	856 (14.34)		325 (6.31)	1,096 (15.00)	
II	701 (5.27)	374 (5.76)	327 (5.48)		249 (4.83)	452 (6.19)	
III	2,783 (22.33)	1,569 (24.15)	1,214 (20.34)		1,215 (23.56)	1,568 (21.45)	
IV	7,559 (60.65)	3,988 (61.39)	3,571 (59.84)		3,367 (65.30)	4,192 (57.36)	
Pathological diagnosis				<0.0001			0.1106
Non small cell lung cancer	6,473 (51.93)	3,296 (50.74)	3,177 (53.23)		2,726 (52.87)	3,747 (51.27)	
Small cell lung cancer	3876 (31.10)	2,198 (33.84)	1,678 (28.12)		1,593 (30.90)	2,283 (31.23)	
Unknown	2,115 (16.97)	1,022 (15.42)	1,113 (18.65)		837 (16.23)	1,278 (17.50)	

### Health Insurance and Lung Cancer Treatments

Table 2 shows the relationship between different medical insurance types and self-paying rate groups and the choice of lung cancer treatment (including surgery, targeted therapy, immunotherapy, radiotherapy, chemotherapy), with treatment mode as the outcome, insurance type and co-payment rate grouping as independent variables, and other adjustment variables were included in the model. After adjusting the age at diagnosis, sex, ethnicity, marital status, occupation, date of diagnosis, cancer stage, and pathological diagnosis, patients receiving UEBMI are more likely choose surgery, targeted therapy, immunity therapy, and radiotherapy, and chemotherapy, and the difference is statistically significant. Compared with patients whose self-paying is <50%, patients with a higher self-paying tend to choose surgery, targeted therapy, immunity therapy and chemotherapy, and the possibility of choosing radiotherapy is unlikely.

**Table 2:** Associations of insurance type and self-paying rate with treatment type.

				**Model A**		**Model B**	
	**Number of patients**	**Number of events**	**%**	**OR (95% CI)**	***P***	**OR (95% CI)**	***P***
Surgery^a^							
By insurance type^b^							
URBMI	6,496	1,570	24.17	1.00		1.00	
UEBMI	5,968	1,714	28.72	1.336 (1.232,1.450)	<0.0001	1.179 (1.077,1.292)	0.001
By self-paying rate^c^							
0%-49%	5,156	946	18.35	1.00		1.00	
50%−100%	7,308	2,338	31.99	1.881 (1.715,2.063)	<0.0001	1.594 (1.441,1.764)	<0.0001
Targeted therapy^a^							
By insurance type^b^							
URBMI	6,496	1,053	16.21	1.00		1.00	
UEBMI	5,968	1,287	21.57	1.506 (1.373,1.653)	<0.0001	1.713 (1.551,1.891)	<0.0001
By self-paying rate^c^							
0%-49%	5,156	826	16.02	1.00		1.00	
50%-100%	7,308	1,514	20.72	1.166 (1.053,1.292)	0.003	1.138 (1.020,1.270)	0.020
Immunity therapy^a^							
By insurance type^b^							
URBMI	6,496	233	3.57	1.00		1.00	
UEBMI	5,968	258	4.32	1.271 (1.057,1.530)	0.011	1.449 (1.200,1.749)	<0.0001
By self-paying rate^c^							
0%-49%	5,156	30	0.58	1.00		1.00	
50%-100%	7,308	461	6.31	8.093 (5.517,11.872)	<0.0001	6.747 (4.583,9.932)	<0.0001
Radiotherapy^a^							
By insurance type^b^							
URBMI	6,496	1,696	26.11	1.00		1.00	
UEBMI	5,968	2,089	35.00	1.537 (1.422,1.662)	<0.0001	1.723 (1.588,1.869)	<0.0001
By self-paying rate^c^							
0%-49%	5,156	2,260	43.83	1.00		1.00	
50%-100%	7,308	1,525	20.88	0.326 (0.298,0.355)	<0.0001	0.358 (0.327,0.391)	<0.0001
Chemotherapy^a^							
By insurance type^b^							
URBMI	6,496	3,007	46.29	1.00		1.00	
UEBMI	5,968	2,850	47.75	1.062 (0.989,1.141)	0.099	1.191 (1.105,1.283)	<0.0001
By self-paying rate^c^							
0%-49%	5,156	2,613	50.68	1.00		1.00	
50%-100%	7,308	3,244	44.39	1.293 (1.196,1.397)	<0.0001	1.246 (1.148,1.352)	<0.0001

*Model A, we adjusted for demographic factors, including age at diagnosis, sex, ethnicity, marital status, and occupation*.

*Model B, based on model A, was also adjusted according to the clinical characteristics, including the date of diagnosis of lung cancer, cancer stage, and pathological diagnosis*.

a *refers to the model with treatment as the outcome*.

bc *refers to insurance type and self-paying rate group are included in the model respectively*.

### Health Insurance and Lung Cancer-Specific Mortality

**Figure 1:**
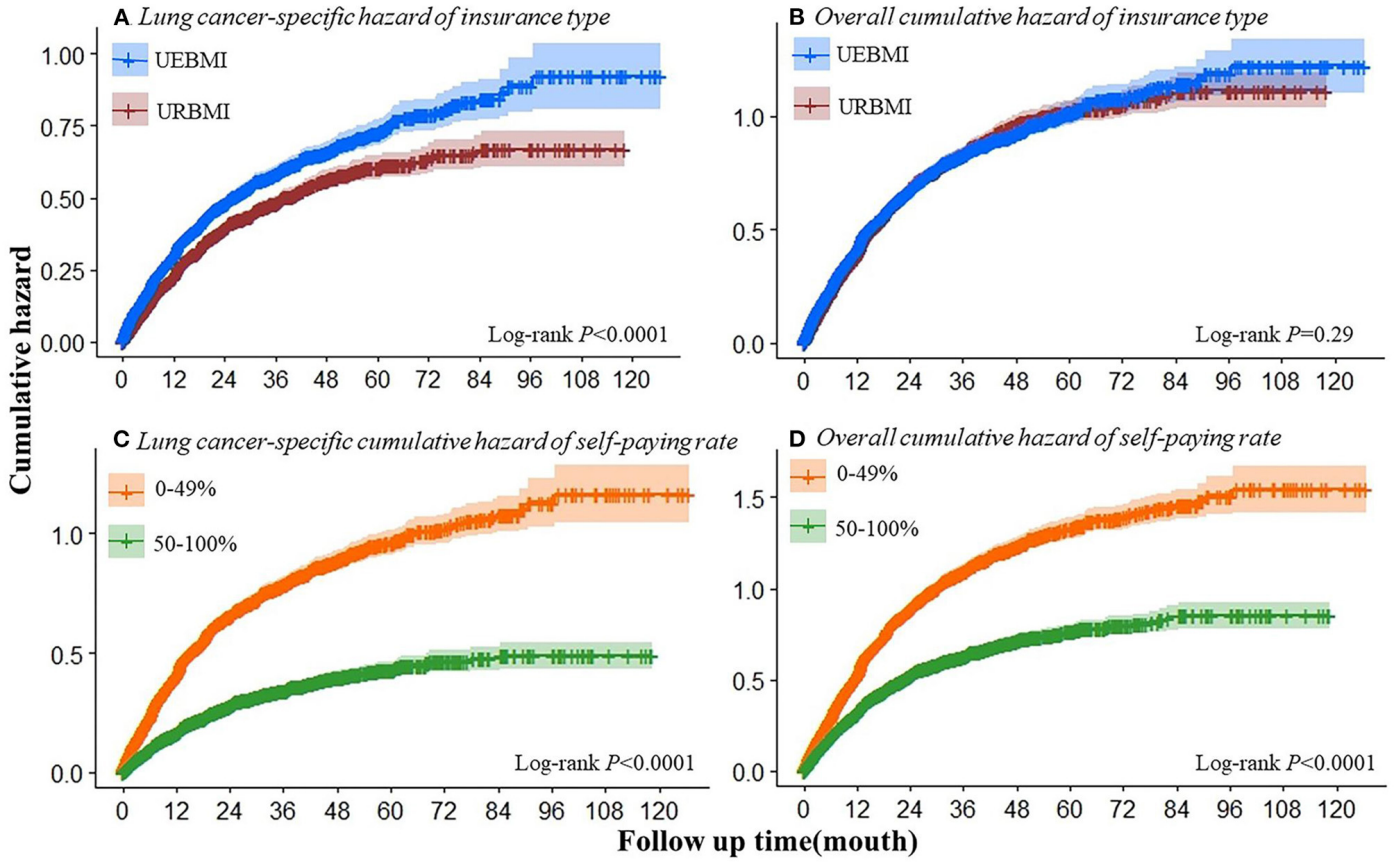
Cumulative hazard of cancer-specific or overall mortality by insurance type **(A,B)** and self-paying rate **(C,D)**.

During the follow-up period (the median follow-up time was 13 months, the quartile range was 5.6–25.2 months), 5,803 deaths were observed, of which 3,781 died of lung cancer. As can be seen in Figure 1, patients receiving UEBMI and patients with self-paying rate had a higher cumulative hazard lung cancer-specific compared with patients receiving URBMI and patients with higher self-paying rate. This group of URBMI patients has a higher self-paying rate and therefore has a lower cumulative hazard of lung cancer relative to UEBMI. The all-cause mortality rate has the same result. When adjusting for demographic characteristics, the risk of lung cancer-specific mortality was increased by 21.6% (95%CI: 14.0%-29.7%) for patients receiving UEBMI compared with patients receiving URBMI (Table 3). After adjusting the clinical characteristics and lung cancer treatment modes, the risk of lung cancer-specific death increased by 38.1% for patients receiving UEBMI (Hazard Ratios (HRs) = 1.381, 95% confidence interval (CI): 1.293–1.476, *P* < 0.005); the risk of all-cause death increased by 14.7% (HRs = 1.147, 95%CI: 1.088–1.1210, *P* < 0.005). Compared with patients with insufficient self-paying rate, the risk of lung cancer-specific death decreased by 40.2% in patients with a higher self-paying rate (HRs = 0.598, 95%CI: 0.557–0.643, *P* < 0.005), and the risk of all-cause death decreased by 30.4% HRs = 0.696, 95%CI: 0.657 ~ 0.737, *P* < 0.005).

**Table 3:** Associations of insurance type and self-paying rate with lung cancer-specific death and all-cause death.

				**Model A^**a**^**		**Model B^**b**^**		**Model C^**c**^**	
	**Number of patients**	**Number of events**	**Rate**	**HR (95%CI)**	***P***	**HR (95% CI)**	***P***	**HR (95% CI)**	***P***
Lung cancer-specific mortality									
By insurance type									
URBMI	6,496	1,731	8.73	1.00		1.00		1.00	
UEBMI	5,968	2,050	9.02	1.216 (1.140,1.297)	<0.0001	1.260 (1.181,1.345)	<0.0001	1.381 (1.293,1.476)	<0.0001
By self-paying rate									
0%-49%	7,308	1,419	5.17	1.00		1.00		1.00	
50%-100%	5,156	1,215	8.05	0.522 (0.487,0.559)	<0.0001	0.618 (0.576,0.662)	<0.0001	0.598 (0.557,0.643)	<0.0001
Overall mortality									
By insurance type									
URBMI	6,496	2,959	8.84	1.00		1.00		1.00	
UEBMI	5,968	2,844	8.96	0.989 (0.939,1.042)	0.687	1.042 (0.989.1.098)	0.122	1.147 (1.088,1.210)	<0.0001
By self-paying rate									
0%-49%	5,156	3,169	8.25	1.00		1.00		1.00	
50%-100%	7,308	2,634	9.84	0.649 (0.614,0.685)	<0.0001	0.715 (0.676,0.756)	<0.0001	0.696 (0.657,0.737)	<0.0001

*Model A, we adjusted for demographic factors, including age at diagnosis, sex, ethnicity, marital status, and occupation*.

*Model B, based on model A, was also adjusted according to the clinical characteristics, including the date of diagnosis of lung cancer, cancer stage, and pathological diagnosis*.

*Model C, based on model B, we additionally adjusted for treatment types, whenever applicable, including surgery, targeted therapy, immunity therapy, radiotherapy, and chemotherapy*.

### Joint Effect of Insurance Type and Self-Paying Rate

Table 4 summarizes the joint effect of insurance type and self-paying rate. For every 10% increase in the self-paying rate, the overall risk of lung cancer-specific death decreases by 12.3% (after complete adjustment, HRs = 0.877, 95%CI: 0.864–0.891, *P* < 0.005), the risk of lung cancer-specific death for patients receiving URBMI was reduced by 17.6% (HRs = 0.896, 95%CI: 0.872–0.921, *P* < 0.005), and the risk of lung cancer-specific death for receiving UEBMI was reduced by 18.0% (HRs = 0.820, 95%CI: 0.797-0.843, *P* < 0.005). Similar results were found in all-cause mortality.

**Table 4:** Association of every 10% insurance self-paying rate increase with lung cancer-specific death and all-cause death.

			**Model A^**a**^**		**Model B^**b**^**		**Model C^**c**^**	
	**Number of patients**	**Number of** **events**	**HR (95%CI)**	***P***	**HR (95% CI)**	***P***	**HR (95% CI)**	***P***
Lung cancer-specific mortality								
Any insurance type									
Per 10% increase	12,464	3,781	0.847 (0.835,0.859)	<0.0001	0.886 (0.873,0.899)	<0.0001	0.877 (0.864,0.891)	<0.0001
Within URBMI									
Per 10% increase	6,496	1,731	0.896 (0.874,0.918)	<0.0001	0.932 (0.909,0.956)	<0.0001	0.896 (0.872,0.921)	<0.0001
Within UEBMI									
Per 10% increase	5,968	2,050	0.758 (0.740,0.777)	<0.0001	0.807 (0.785,0.829)	<0.0001	0.820 (0.797,0.843)	<0.0001
Overall mortality								
Any insurance type								
Per 10% increase	12,464	5,803	0.913 (0.902,0.923)	<0.0001	0.933 (0.922,0.944)	<0.0001	0.923 (0.912,0.934)	<0.0001
Within URBMI									
Per 10% increase	6,496	2,959	0.951 (0.933,0.969)	<0.0001	0.970 (0.952,0.989)	0.002	0.936 (0.916,0.956)	<0.0001
Within UEBMI									
Per 10% increase	5,968	2,844	0.831 (0.816,0.847)	<0.0001	0.863 (0.845,0.881)	<0.0001	0.883 (0.864,0.902)	<0.0001

*Model A, we adjusted for demographic factors, including age at diagnosis, sex, ethnicity, marital status, and occupation*.

*Model B, based on model A, was also adjusted according to the clinical characteristics, including the date of diagnosis of lung cancer, cancer stage, and pathological diagnosis*.

*Model C, based on model B, we additionally adjusted for treatment types, whenever applicable, including surgery, targeted therapy, immunity therapy, radiotherapy, and chemotherapy*.

## Discussion

This study uses the HIS and follow-up database to comprehensively analyze the relationship between specific deaths and all-cause deaths in Chongqing lung cancer patients and insurance types or self-paying rate. Based on the 12,464 lung cancer patients treated in the Cancer Hospital of Chongqing from 2013 to 2020, the subgroup analysis was carried out according to insurance type and self-paying rate. Our results showed that the higher self-paying rate is related to the risk of lung cancer-specific death, the reduction is significantly correlated. In the study of the joint effect of the medical insurance type and self-paying rate, we found that every 10% increase in self-paying rate of URBMI reduces the risk of lung cancer-specific death by 17.6%, while every 10% increase in self-paying rate of UEBMI reduces the risk of lung cancer-specific death by 18.0%.

With the continuous development of medical and health services, the research and development of drugs for the treatment of lung cancer can prolong the survival time of patients, but at the same time it also further increases the economic burden of patients and their families. Since most anti-cancer drugs were not included in the medical insurance list in the past 10 years, and most of the targeted anti-cancer drugs in China are imported drugs, they are affected by factors such as excessive tariffs, many circulation links, and few suppliers, resulting in overprice of anti-cancer drugs (16–19). If lung patients want to extend their survival time and improve their survival status, they must bear the high cost themselves. This explains the increase in self-paying rate of lung cancer patients in this study, and the reduced risk of death. But often want to reduce mortality, lung cancer patients and their families need to bear a great economic burden.

Fortunately, this phenomenon has been discovered by China's medical insurance management department. Since 2006, the Chinese government has issued many policies to reduce the financial burden of lung cancer patients, such as the price of anti-cancer drugs and critical illness insurance (20). At present, more and more anti-cancer drugs are included into insurance. Especially in December 2021, the National Medical Security Administration announced that 74 new drugs were included in the national medical insurance catalog, of which 7 directly added drugs including a drug for the treatment of cancer (eg. Bendamustine). The 67 newly negotiated drugs include 11 anti-tumor drugs, of which 3 drugs (eg. Ensartinib Hydrochloride capsules, Almonertinib Mesilate Tablets, and Dacomitinib Tablets) treat non-small cell lung cancer. It can be seen that, this measure not only reduces the medical burden of lung cancer patients, but also enables more lung cancer patients to use expensive drugs, so as to reduce the mortality of lung cancer, which is undoubtedly a good thing for patients and their families. In addition to including anti-cancer drugs as much as possible into the medical insurance list, early screening for people at high risk of lung cancer is also particularly important (21, 22). The clinical symptoms of lung cancer patients are not particularly obvious. Once discovered, most patients (62%) have basically reached the advanced stage of lung cancer. Studies have shown that more than 90% of the total medical expenses occur in the year after the diagnosis of lung cancer, and long-term treatment will not only have bad results but also bring a heavy financial burden (23, 24). According to the characteristics of the population in my country, high-risk groups of lung cancer should undergo low-dose computed tomography screening every year, and positron emission tomography/computed tomography screening methods are not recommended. At present, there is no clear conclusion on the pathogenic factors of lung cancer, but many research scholars have shown that the age over 40 years old and long-term heavy smoking are closely related to the incidence of lung cancer. Smoking not only harms my health, but second-hand smoke also affects the surrounding people. Health is affected, and the number of lung cancer patients among passive smokers is also increasing (25). In addition to smoking, occupational exposure history (asbestos, beryllium, uranium, radon, etc.), family genetic history and other factors can also affect the occurrence of lung cancer (26–29). In daily life, men should quit smoking, women should try to avoid passive smoking and less exposure to oil fume (30); protective measures should be taken for people with occupational exposure risks; when the atmosphere is severely polluted, avoid going out and exercising.

This study has several advantages, because these data are obtained from the cancer hospital follow-up database and the hospital HIS system. It is the first time to analyze the relationship between lung cancer patients and their costs in the past 10 years. In addition, this study can provide certain information to the Chongqing Medical Insurance Bureau and provide ideas for the development of Chongqing and my country's medical insurance. However, this study also has certain limitations. First of all, the cost of treatment of lung cancer patients used in this study is only incurred in the hospital, and the cost incurred outside the hospital cannot be estimated. Second, the lung cancer patients in this study only included patients from Chongqing's 3A-level tumor specialist hospitals, but not patients from other hospitals in Chongqing. Third, this study only discusses the relationship between mortality and insurance type and self-paying rate. Finally, active follow-up of patients in this study may lead to information bias. In future studies, outcome variables should be more detailed, such as patient survival time.

## Conclusions

The results of this study suggest that patients with lower self-paying rate in China face a higher risk of lung cancer-specific deaths. The reason may be that some drugs for the treatment of lung cancer are not covered by medical insurance. If lung cancer patients want to reduce the risk of death and obtain better treatment effects, they can only use these drugs that are not covered by medical insurance, and these drugs are often expensive. This phenomenon has been discovered by China's medical insurance management department. Therefore, in recent years, the National Medical Insurance Bureau is also adjusting the policy. For example, under the condition of limited medical insurance funds, the original self-paid anti-tumor drugs are included in the national medical insurance scope as much as possible, thereby reducing the family economy burden. On the other hand, it also shows that high-risk groups in China should improve their awareness of early screening of lung cancer and actively participate in the national cancer screening project led by the state Declarations.

## Data Availability Statement

The data analyzed in this study is subject to the following licenses/restrictions. The datasets used and/or analyzed during the current study, are available from the corresponding authors on reasonable requests to access these datasets should be directed to YoW, tohongying@163.com.

## Ethics Statement

All procedures involving human participants were in accordance with the ethical standards of the institution or practice at which the study was performed. We followed relevant guidelines to ensure that the study was voluntary and confidential. The authors are accountable for all aspects of the work in ensuring that questions related to the accuracy or integrity of any part of the work are appropriately investigated and resolved.

## Author Contributions

YuW, HL, YiW, YoW, and BP designed the study protocol. YuW and HL performed statistical analysis and interpretation. YuW drafted the manuscript. WZ and XL performed the data collection. YiW and BP participated to the data cleaning. GW and AS revised the article. All authors read and approved the final manuscript.

## Funding

This work was supported by the Chongqing Performance Incentive and Guidance Project for Scientific Research Institutions (cstc2020jxjl130016); Chongqing Key Disease Prevention and Control Technology Project (2019ZX002); Novel Coronavirus Infection and Prevention Emergency Scientific Research Special Project of Chongqing Municipal Education Commission, China (KYYJ202001).

## Conflict of Interest

The authors declare that the research was conducted in the absence of any commercial or financial relationships that could be construed as a potential conflict of interest.

## Publisher's Note

All claims expressed in this article are solely those of the authors and do not necessarily represent those of their affiliated organizations, or those of the publisher, the editors and the reviewers. Any product that may be evaluated in this article, or claim that may be made by its manufacturer, is not guaranteed or endorsed by the publisher.
